# Impact of the acidic environment on gene expression and functional parameters of tumors in vitro and in vivo

**DOI:** 10.1186/s13046-020-01815-4

**Published:** 2021-01-06

**Authors:** Mandy Rauschner, Luisa Lange, Thea Hüsing, Sarah Reime, Alexander Nolze, Marcel Maschek, Oliver Thews, Anne Riemann

**Affiliations:** Institute of Physiology, University Halle-Wittenberg, Magdeburger Str. 6, 06112 Halle (Saale), Germany

**Keywords:** Acidosis, Hypoxia, Gene expression, Migration, Proliferation

## Abstract

**Background:**

The low extracellular pH (pH_e_) of tumors resulting from glycolytic metabolism is a stress factor for the cells independent from concomitant hypoxia. The aim of the study was to analyze the impact of acidic pH_e_ on gene expression on mRNA and protein level in two experimental tumor lines in vitro and in vivo and were compared to hypoxic conditions as well as combined acidosis+hypoxia.

**Methods:**

Gene expression was analyzed in AT1 prostate and Walker-256 mammary carcinoma of the rat by Next Generation Sequencing (NGS), qPCR and Western blot. In addition, the impact of acidosis on tumor cell migration, adhesion, proliferation, cell death and mitochondrial activity was analyzed.

**Results:**

NGS analyses revealed that 147 genes were uniformly regulated in both cell lines (in vitro) and 79 genes in both experimental tumors after 24 h at low pH. A subset of 25 genes was re-evaluated by qPCR and Western blot. Low pH consistently upregulated Aox1, Gls2, Gstp1, Ikbke, Per3, Pink1, Tlr5, Txnip, Ypel3 or downregulated Acat2, Brip1, Clspn, Dnajc25, Ercc6l, Mmd, Rif1, Zmpste24 whereas hypoxia alone led to a downregulation of most of the genes. Direct incubation at low pH reduced tumor cell adhesion whereas acidic pre-incubation increased the adhesive potential. In both tumor lines acidosis induced a G1-arrest (in vivo) of the cell cycle and a strong increase in necrotic cell death (but not in apoptosis). The mitochondrial O_2_ consumption increased gradually with decreasing pH.

**Conclusions:**

These data show that acidic pH_e_ in tumors plays an important role for gene expression independently from hypoxia. In parallel, acidosis modulates functional properties of tumors relevant for their malignant potential and which might be the result of pH-dependent gene expression.

## Background

In comparison to healthy tissue many tumors show pronounced extracellular acidosis with pH values even below 6.0 [1]. These adverse environmental conditions result from increased glycolytic metabolism due to an insufficient oxygen supply. Thus, many tumors are characterized by concomitant hypoxia and extracellular acidosis [2, 3]. But even with sufficient O_2_ supply tumors show intensified glycolysis which has been described as Warburg effect and seems to be characteristic for rapidly dividing cells [4]. The impact of hypoxia per se on gene expression and cell function has been studied extensively in vitro and in vivo and numerous genes related to different functional properties of tumor cells have been shown to be regulated by low tissue pO_2_ often via the hypoxia inducible factor HIF.

Low extracellular pH (pH_e_) has also been shown to modulate tumor cell function. Several studies revealed that acidosis increased the local invasiveness of tumors and the metastatic spread of tumors in vivo [5, 6]. Tumor cells kept at low pH led to a significant higher number of lung metastases [7, 8]. Several mechanisms were discussed to be causative for the increased metastatic potential. Besides the induction of angiogenesis in acidotic tumors [9] or a pH-dependent degradation of the extracellular matrix [8, 10] tumor cell migration is increased at moderately acid pH [7, 11]. Furthermore epithelial-to-mesenchymal transition (EMT) of cells has been shown to be modulated by acidosis [12, 13]. Besides the metastatic spread, the sensitivity of tumors to non-surgical treatment modalities was found to be pH-dependent. The efficacy of chemo- as well as of immunotherapy has been shown to be sensitive to an extracellular acidosis [14, 15]. Different mechanisms have been discussed by which the H^+^-level may affect the sensitivity, for instance, by affecting the drug permeability of the cell membrane [16], by the activation of active drug efflux pumps [17] or by acidosis-dependent changes of cytokine expression of tumor-associated immune cells [16, 18].

Many of these functional changes can be achieved by alterations of gene expression. For instance, it was shown that tumor cells change the expression of inflammatory cytokines (e.g., MCP-1, iNOS) at moderately low pH [19]. Also, the expression of matrix degrading enzymes (e.g., MMP-9) is acidosis-dependent [20]. However, most of these expression studies were performed only on a limited number of target genes and only the impact of isolated acidosis was analyzed. In tumors in vivo acidosis is always accompanied by a low pO_2_ (hypoxia) since the acidification results i.a. from the glycolytic metabolism induced by the O_2_ deficiency.

For these reasons, the aim of this study was to analyze the gene expression on the mRNA and protein level in two different tumor lines in isolated tumor cells under acidic or hypoxic conditions and during combined acidosis+hypoxia. These data were compared with in vivo expression results of experimental tumors (of the same tumor lines) in control tumors and in tumors in which a more pronounced tumor acidosis was induced either by forcing the glycolytic metabolism (inspiratory hypoxia + uncoupling the respiratory chain) or by intratumoral acid injection. In order to study the functional consequences of the altered expression functional parameters like proliferation, cell cycle distribution, apoptosis, necrosis, cell migration and cell adhesion were analyzed under the acidic conditions. Finally, the impact of extracellular acidosis on the activity of the different steps of the respiratory chain within the mitochondria was measured.

## Material and methods

### Cell lines

All studies were performed with two tumor cell lines of the rat: (a) subline AT1 of the Dunning prostate carcinoma R3327 (CLS # 500121, CLS GmbH, Eppelheim, Germany) and (b) Walker-256 mammary carcinoma (ATCC # CCL-38, LGC Standards GmbH, Wesel, Germany). The AT1 subline was established from the R3327 Dunning carcinoma cell line which was discovered in a male Copenhagen rat. The AT1 line is a anaplastic tumor growing androgen-independently [21]. In vivo AT1 cells form undifferentiated, densely packed tumors (without glandular structures) with a volume doubling time during the exponential growing phase of about 2.2 days and low metastatic potential [21]. AT1 cells show strong adhesion to collagen IV, laminin and natural extracellular matrix leading to low invasive potential [22]. The cell population shows a high amount of aneuploidic cells which highly express adhesion markers like CD24, CD44, CD326 or cytokeratin 19 [23].

The Walker-256 cell line was developed from a spontaneous tumor in the mammary gland of a pregnant rat and has been described as a carcinosarcoma [24]. The cells are undifferentiated but from the morphological aspect two distinct cell types could be classified. However, it is unclear whether the morphological differences result from independent cell types or whether they result from a multipotential stem cell [24]. Walker-256 cells are rapidly dividing and are tumorigenic after subcutaneous injection. The resulting tumors are undifferentiated [25] and grow with a volume doubling time of about 1.7 days. In contrast to the AT1 tumor model, Walker-256 tumor cells are highly metastatic forming bone and brain metastases [25, 26] during which a active destruction of subendothelial matrix by metalloproteinases plays a role [27].

Both cell lines were cultured in RPMI medium supplemented with 10% fetal calf serum (FCS) and for Walker-256 cells additionally with 10 mM L-glutamine, 20 mM HEPES, 7.5% NaHCO_3_. For the experiments, cells were incubated under serum starvation for 24 h either in medium buffered with NaHCO_3_, 10 mM MES (morpholinoethanesulfonic acid) and 10 mM HEPES, pH adjustment to pH 7.4 or 6.6 with 1 N HCl. Normoxic culture conditions were obtained by incubating the cells with room air containing 5% CO_2_. Hypoxic culture conditions were induced in a hypoxia chamber (HypoxyLab, Oxford Optronix, Oxford, UK) at 0.2% O_2_ (pO_2_ = 1.5 mmHg) and 5% CO_2_. For analysis of mRNA and protein expression cells were harvested and lysed after 24 h incubation under the respective conditions.

### In vivo tumor models

Solid tumors of AT-1 cells were induced in vivo in male Copenhagen rats (body weight 145–332 g) and Walker-256 tumors in Wistar rats (body weight 213–284 g), housed in the animal care facility of the University of Halle. All experiments had previously been approved by the regional animal ethics committee and were conducted in accordance with the German Law for Animal Protection and the UKCCCR Guidelines [28].

Solid tumors were induced by heterotopic injection of cell suspension (6-8 × 10^6^ cells/0.4 ml isotonic saline) subcutaneously into the dorsum of the hind foot. Tumor volumes were determined by measuring the three orthogonal diameters with a caliper and with the formula: V = d_1_·d_2_·d_3_·π/6. Tumors were investigated when they reached a volume of 0.35–1.50 ml.

In order to induce a more pronounced tumor acidosis in vivo, two different methods were used. Firstly, metabolic acidosis was induced by treating tumor-bearing animals with a combination of inspiratory hypoxia and meta-iodobenzylguanidine (MIBG) which forces glycolytic metabolism [29]. Therefore, animals received a MIBG injection (20 mg/kg b.w., i.p. dissolved in isotonic saline) and were then housed in a hypoxic atmosphere containing 10% O_2_ and 90% N_2_ for 24 h. This procedure reduces the extracellular pH in AT1 tumors from 7.02 ± 0.04 to 6.48 ± 0.08 and in Walker-256 tumors from 7.16 ± 0.03 to 6.65 ± 0.07 [30]. Animals housed in room air receiving only the solvent served as control. Secondly, tissue acidosis was intensified by direct intratumoral injection of lactic acid. Therefore, 50 μl of 0.222 mM lactic acid (in H_2_O) were injected into the tumor tissue at a depth of 2–3 mm. Treatment of the contralateral tumor with 50 μl of 0.222 mM sodium lactate served as intra-individual control.

After 24 h animals were sacrificed, tumors were surgically removed, minced and total RNA was extracted using TRIzol reagent (Thermo Fisher Scientific, Waltham, MA, USA), while protein was isolated by using CST cell lysis buffer (20 mM Tris-HCl (pH 7.5), 150 mM NaCl, 1 mM EDTA, 1 mM EGTA, 1% Triton, 2,5 mM sodium pyrophosphate, 1 mM ß-glycerophosphate, 1 mM Na_3_VO_4_, protease inhibitor cocktail).

### mRNA expression

For mRNA expression analyses total RNA was isolated using TRIzol according to the manufacturer’s instructions. mRNA expression in cells and tumors was assessed by Next Generation-Sequencing followed by validation of the expression using quantitative PCR. Sequencing was performed on an Illumina System by Novogene Co Ltd. (Hongkong), followed by a raw data quality check, adapter clipping, quality trimming and alignment against the rat genome (RGSC 6.0/rn6). Initial mapping was done with Bowtie2 followed by Tophat2 using Bowtie to align spliced reads. Finally counting was performed with featureCounts and genes were annotated with BiomaRt v93. Normalization and differential expression analysis were performed using DESeq2 and EdgeR. Samples for NGS were obtained from lysates of cells incubated under acidotic and control conditions as well as from samples of tumors with intensified glycolytic metabolism and controls of both tumor lines. For further analysis mRNAs were eliminated which show low abundance (≤ 10 fragments per million FPM). Fig. S1 (Additional file 1) shows the number of genes fulfilling this criterion in each experimental group. For detectable mRNAs the expression ratio acidosis-to-control was calculated. An acidosis-induced regulation was defined if in cells the expression was up- or downregulated by a factor of 1.5 and in tumors by a factor of 1.75. In order to analyze whether the acidosis-regulated genes may play a relevant role for functional properties of tumor cells (e.g., proliferation rate, cell death, activation of signaling pathways) a gene ontology analysis was performed from the NGS results for those genes which were significantly consistently up- or down regulated in AT1 and Walker-256 tumor cells or solid tumors in vivo after 24 h of extracellular acidosis. This analysis was performed using the PANTHER Classification System (Ver. 15) and the GO-slim Overrepresentation Test [31]. By this, ontologies (biological processes and molecular functions) were identified which were statistically significant overrepresented (with false discovery rate (FDR) correction).

From the complete list of pH-regulated genes 25 (*Acat2, Aox1, App, Brip1, Calcoco1, Clspn, Crem, Dnajc25, Ercc6l, Fstl1, Fundc1, Gls2, Gstp1, Ikbke, Il6r, Lamp2, Ltbp2, Mmd, Per3, Pink1, Rif1, Tlr5, Txnip, Ypel3, Zmpste24*) were selected which were consistently regulated either in both cell lines in vitro or in vivo and which have been described to in literature to play a relevant rule in the malignant progression of tumors. For qPCR validation 1 μg RNA was subjected to reverse transcription with SuperScript II reverse transcriptase (Thermo Fisher Scientific, Waltham, MA, USA) and analyzed by qPCR using the Platinum SYBR Green qPCR Supermix (Thermo Fisher Scientific, Waltham, MA, USA). The obtained data were normalized against *18S* or *Hprt1*, which are suitable housekeeping genes for studying tumor acidosis [32], and were related to the respective control. Suppl. Tab. S1 shows the primers used.

### Western blot

Western blotting was performed according to standard protocols. In brief, cells were lysed (0.5 M Tris-HCl pH 6.8; 10% SDS; 10% 2-mercaptoethanol; 20% glycerol; 0.01% bromophenol blue), separated by sodium dodecyl sulfate polyacrylamide gel electrophoresis, and transferred to a nitrocellulose membrane. Subsequently, membranes were incubated with antibodies specific for CREM (#PA5–29927, Invitrogen, Darmstadt, Germany), GLS2 (#PA5–78475, Invitrogen), PER3 (#PA5–40922, Invitrogen) and TXNIP (#14715, Cell Signaling, Danvers MA, USA). The bound primary antibody was visualized by IRDye secondary antibodies (Licor Biosciences, Lincoln, NE, USA) with the imaging system Odyssey (Licor Biosciences, Lincoln, NE, USA). Quantitative analysis was performed with Image Studio Lite software (Licor Biosciences, Lincoln, NE, USA).

### Tumor cell migration

The migratory speed of AT1 tumor cells was determined after 24 h incubation at pH 7.4 or 6.6. For time lapse microscopy 6 × 10^5^ cells were grown in 35 mm-Petri dishes, incubated with the buffers at different pH and transferred to an incubation chamber (stage Top Incubator INU-KI-F1; Tokai Hit) of a Keyence BZ-8100E fluorescence microscope (Keyence, Osaka Japan). Cell migration was measured over a time interval of 100 min with imaging every 5 min. Single cells were tracked in this series of 20 images and the averaged migratory speed (in μm/min) as well as the covered distance (in μm) was determined. For the calculations ImageJ software (ibidi Chemotaxis and Migration Tool, Gräfelfing, Germany) was used.

### Wound closure assay

Migration was also assessed by a wound closure assay (Scratch Assay) using an automated video analysis system (IncuCyte Scratch Wound Migration and Invasion Assay, Essen BioScience, Ann Arbor MI, USA). The experiments were performed in accordance to the manufacturer’s instructions. In brief, AT1 cells were cultured in 96-well plates (1 × 10^5^ cells/well). 24 h before the measurement medium was replaced to fresh medium (pH 7.4) without FCS and after 18 h medium was replaced by buffer with the respective pH (7.4 or 6.6). After 3 h incubation a defined wound area was created using Essen 96-well WoundMaker and the 96-well plate was transferred to an incubator for 24 h in which the closure of the wound was followed by a video system. Wound width (in μm) and the percentage of wound closure was calculated.

### Cell adhesion

Cell adhesion was measured by continuous impedance measurements of monolayer cells (xCELLigence DP; OLS OMNI Life Science, Bremen, Germany) in accordance to the manufacturer’s instructions. First, it was tested whether cells lose their adherence if they are exposed to low pH. Therefore, cells were plated on 16-well plates for 48 h to establish a tight contact between cells and plate surface. Thereafter, medium was changed to pH 7.4 or pH 6.6 and impedance was followed for 48 h. In the second series it was tested whether priming the cells at low pH for 24 h will affect the ability to adhere on the surface. Therefore, cells were pre-incubated at pH 6.6 or 7.4 in normal petri dishes for 24 h. Subsequently, cells were mechanically detached and the cell suspensions were then transferred to 16-well plates in which the impedance was measured during the next 48 h.

### Cell cycle distribution and proliferation

For analysis of DNA content and the fraction of actively DNA-synthesizing cells, cells were incubated with 5 μM BrdU (Bromodeoxyuridine) for 1 h. Cells were then fixed with 70% ethanol and stained with anti-BrdU-antibody or isotype control (BD Biosciences, San Jose, CA, USA) and secondary anti-mouse-FITC-antibody (1:100) (Rockland, Limerick, PA, USA). Additionally, cells were stained for 10 min with 50 μg/ml propidium iodide+RNase to measure cell cycle distribution. For analyses of tumors, BrdU was dissolved in PBS and injected i.p. (150 mg/kg body wt). After 120 min, tumors were excised and mechanically disintegrated into a single cell suspension.

### Apoptosis and necrosis

Caspase-dependent apoptosis was assessed by measuring the activity of the effector caspase-3 as described previously. In brief, cells were lysed, centrifugated and the supernatant was incubated with DEVD-AFC. The fluorescence of the cleaved dye 7-amino-4-trifluoromethylcoumarin (AFC) was measured in a multiwell counter (Infinite, Tecan, Berlin, Germany). Protein content was determined with Pierce BCA protein assay (Thermo scientific, Waltham, MA, USA) using bovine serum albumin as standard. For measurements in tumor samples small tissue specimens were minced before lysis. Necrosis in cultured cells was measured by LDH release. LDH activity in media and in cell lysates was measured using standard protocol adapted to lower scale (200 μl).

### Cellular oxygen consumption

The Seahorse XFe96 analyzer (Agilent, Santa Clara CA, USA) was used to measure the oxygen consumption rate by following the decrease in dissolved oxygen in sealed wells of a 96-well plate. In order to analyze different steps of mitochondrial O_2_ metabolism different inhibitors of the respiratory chain were added (XF Cell Mito Stress Test Kit, Agilent). By adding oligomycin (inhibiting complex V ATP synthase) O_2_ use for ATP production can be calculated. Adding carbonyl cyanide-4-(trifluoromethoxy) phenylhydrazone (FCCP; uncoupling oxygen consumption from ATP production) reveals the maximal respiration rate and rotenone + antimycin A incubation (inhibiting complexes I and III) leads to the non-mitochondrial O_2_ consumption.

### Statistical analysis

Results are expressed as means±SEM. Differences between groups were assessed by the two-tailed t-test for paired and unpaired samples. The significance level was set at α = 5% for all comparisons.

## Results

### mRNA and protein expression

**Fig. 1:**
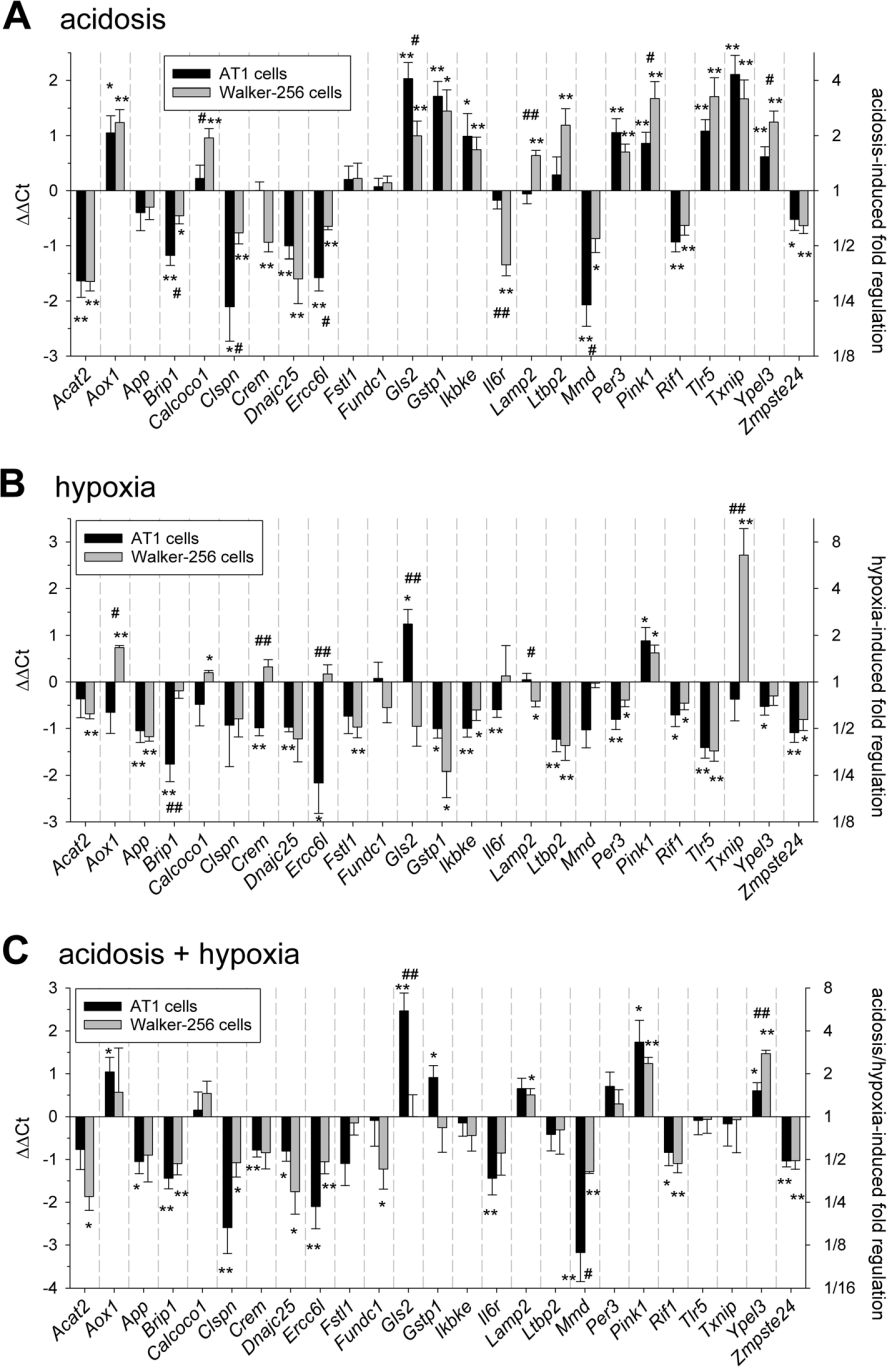
mRNA expression of tumor-associated genes (measured by qPCR) in AT1 prostate and Walker-256 mammary carcinoma cells after 24 h under (**a**) acidotic (pH 6.6, room air), (**b**) hypoxic (pH 7.4, pO_2_ = 1.5 mmHg) or (**c**) combined acidotic and hypoxic (pH 6.6, pO_2_ = 1.5 mmHg) conditions. *n* = 4–27; (*) *p* < 0.05, (**) *p* < 0.01 vs. control; (^#^) p < 0.05, (^##^) p < 0.01 AT1 vs. Walker-256 cells

NGS analyses were performed in both tumor lines in cultured cells and in experimental tumors. Following the criteria mentioned above, 703 (AT1 cells), 1350 (AT1 tumors), 1184 (Walker-256 cells) and 1099 (Walker-256 tumors) genes were regulated, respectively, under acidotic conditions for 24 h. In order to identify cell line independent effects further analyses were only performed on genes which were uniformly regulated (either up or down) in both cell lines (136 genes) or tumors entities (287 genes, Suppl. Fig. S1). From these genes a subset of 25 was selected which have been described in the literature to play a relevant role in the malignant progression of tumors (e.g., Crem, Fstl1, Txnip). Figure 1a shows the impact of acidosis on gene expression in cultured cells (measured by qPCR). Most of the genes were consistently regulated in both tumor cells lines either up or down (for comparison NGS results are shown in Suppl. Fig. S2). If the cells were kept 48 h under acidic conditions the mRNA changes were even more pronounced than after 24 h (Suppl. Fig. S3). For instance, Txnip expression in AT1 cells, which was increased by a factor of 4 after 24 h, was almost 32-times higher after 48 h.

**Fig. 2:**
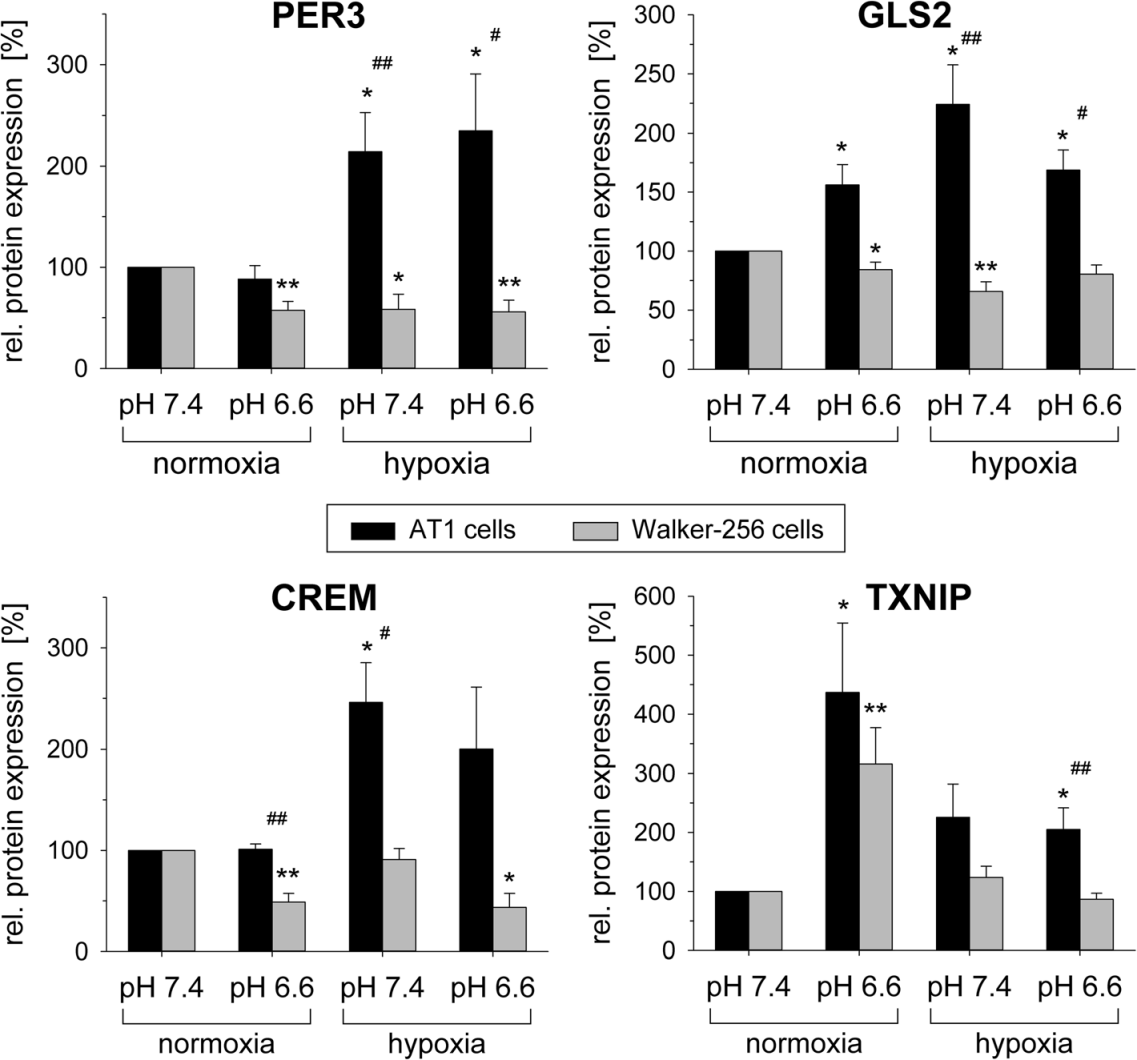
Change of protein levels of PER3, GLS2, CREM and TXNIP in AT1 prostate and Walker-256 mammary carcinoma cells after 24 h under acidotic (pH 6.6) and/or hypoxic (pO_2_ = 1.5 mmHg) conditions. n = 4–12; (*) p < 0.05, (**) p < 0.01 vs. control; (^#^) p < 0.05, (^##^) p < 0.01 AT1 vs. Walker-256 cells

In contrast to acidosis, hypoxia (pO_2_ = 1.5 mmHg) at normal pH significantly reduced the expression of almost all of these 25 genes in both cell lines (Fig. 1b). In order to analyze the situation which corresponds to the in vivo situation, expression in cells was also measured under the simultaneous combination of acidosis+hypoxia (Fig. 1c). For many genes, the overall effect of combined acidosis and hypoxia reflected an additive impact of both conditions. For instance, the expression of Tlr5 (toll like receptor 5) was increased by acidosis and significantly downregulated by hypoxia, the combination of both had almost no impact on the mRNA level (Fig. 1c). Protein expression analyses of the tumor relevant genes CREM GLS2, PER3 and TXNIP mostly showed a diametrical regulation compared to mRNA expression in both cell lines under acidotic conditions (Fig. 2). For example, GLS2 (glutaminase 2) protein was significantly upregulated in AT1 cells but slightly downregulated in Walker-256 cells even though mRNA expression was doubled in these cells (Fig. 1a). Only TXNIP expression increased at low pH on mRNA and protein level in both cell lines.

**Fig. 3:**
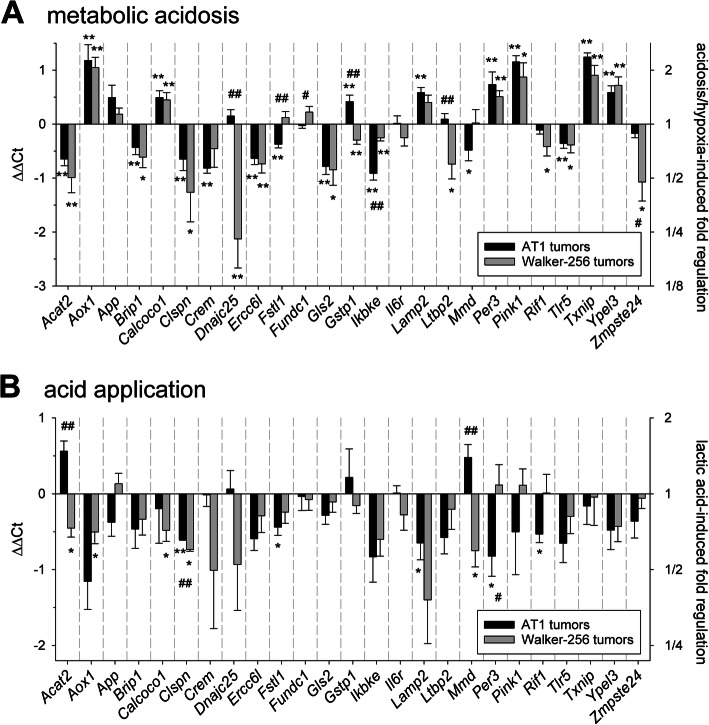
mRNA expression of tumor-associated genes (measured by qPCR) in AT1 prostate and Walker-256 mammary carcinoma tumors in vivo 24 h after (**a**) inducing metabolic acidosis by forcing glycolytic metabolism or (**b**) intratumoral injection of lactic acid. n = 4–22; (*) p < 0.05, (**) p < 0.01 vs. control; (^#^) p < 0.05, (^##^) p < 0.01 AT1 vs. Walker-256 tumors

**Fig. 4:**
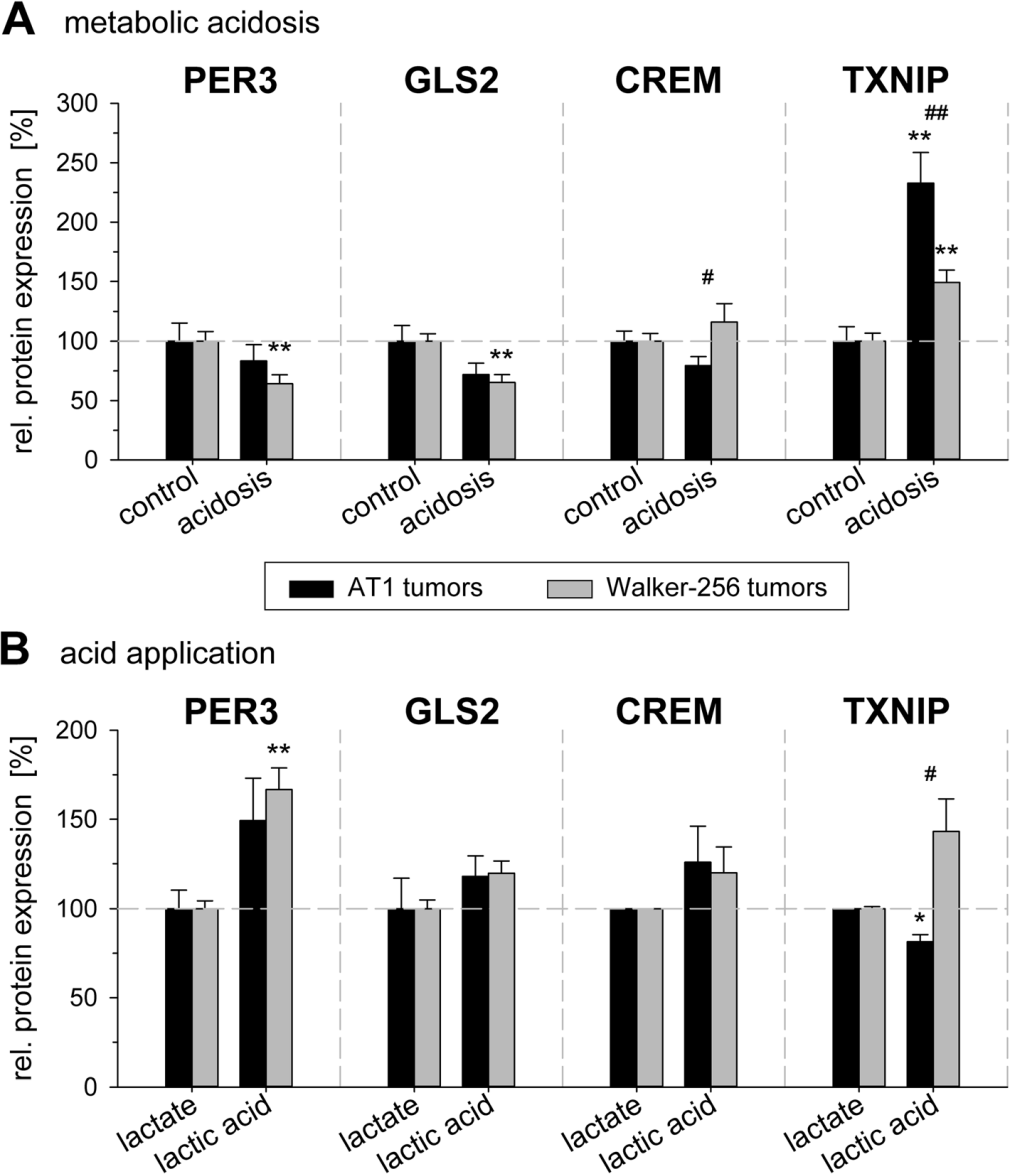
Protein levels of PER3, GLS2, CREM and TXNIP in AT1 prostate and Walker-256 mammary carcinoma tumors in vivo 24 h after (**a**) inducing metabolic acidosis by forcing glycolytic metabolism or (**b**) intratumoral injection of lactic acid. n = 4–18; (*) p < 0.05, (**) p < 0.01 vs. control; (^#^) p < 0.05, (^##^) p < 0.01 AT1 vs. Walker-256 cells

To assess the impact of extracellular acidification in solid tumors two different techniques were used. Glycolytic metabolism was stimulated by inspiratory hypoxia and uncoupling the respiratory chain. Since not only the pH was lowered but also the tissue pO_2_ was reduced, these conditions were comparable to the cell experiments in which acidosis and hypoxia were combined (Fig. 1c). Figure 3a shows the results concerning mRNA expression. Most of the genes were downregulated in both tumor lines. Only Lamp2, Per3 and Txnip showed a significant increase in mRNA expression. On protein level TXNIP expression increased in both tumor lines after 24 h (Fig. 4a). As a second method to lower the tumor pH locally, a direct injection of small amounts of lactic acid was used and the results were compared to tumors in which the same amount of Na^+^-lactate was injected. Using this method mRNA expression of almost all genes was downregulated in both tumor entities (Fig. 3b), however, due to large inter-tumor variability these changes were not statistically significant. On the protein level (Fig. 4) only PER3 was upregulated in Walker-256 tumor.

Recent studies reported that tumor cells adapt to long-term acidosis. Exposing tumor cells to low pH for 4 weeks or longer induced changes in protein expression and functional properties [33–36]. In order to analyze whether the changes in gene expression described above are the result of a short-term acidification (24 h) or can also be found in tumor cells chronically adapted to low pH, AT1 cells were kept for 11 passages (5 weeks) at pH 6.6. Thereafter gene expression was analyzed by NGS and compared to control conditions as well as to acutely acidotic tumor cells. In the first passages after changing to low pH medium, proliferation of the AT1 cells was markedly slowed down, but in the following passages the cell doubling became faster, however, not reaching the control level (Suppl. Fig. S7). After 5 weeks of acidosis the expression of a large number of genes was changed in AT1 cells. Compared to acute acidosis (24 h) the number of pH-regulated genes was approximately 5-times higher after 5 weeks (Suppl. Fig. S8A), 1631 genes were upregulated and 1598 genes were downregulated. A direct comparison of the genes of interest shown in Fig. 1 revealed that several genes were regulated uniformly after 24 h and 5 weeks, such as Fundc1, Gls2, Per3 or Tlr5 (Suppl. Fig. S8B). However, several genes showed an opposite regulation, such as Acat2, Brip1 or Ercc6l. Txnip for instance, which was found to be strongly upregulated after 24 h was almost not regulated after long-term acidosis adaptation.

Several genes found to be regulated by acidosis after 24 h has been described to affect functional parameter of tumor progression, such as proliferation (e.g., Gls2 [37]), cell death (e.g., Txnip, Gls2 [38, 39] tumor cell migration (e.g., Per3, Ikbke, Txnip [40–42] and adhesion (e.g., Txnip [43]) or mitochondrial activity (e.g., Txnip [44]). Therefore the impact of acidification on these cell functions was analyzed in the tumor models.

### Functional parameters

**Fig. 5:**
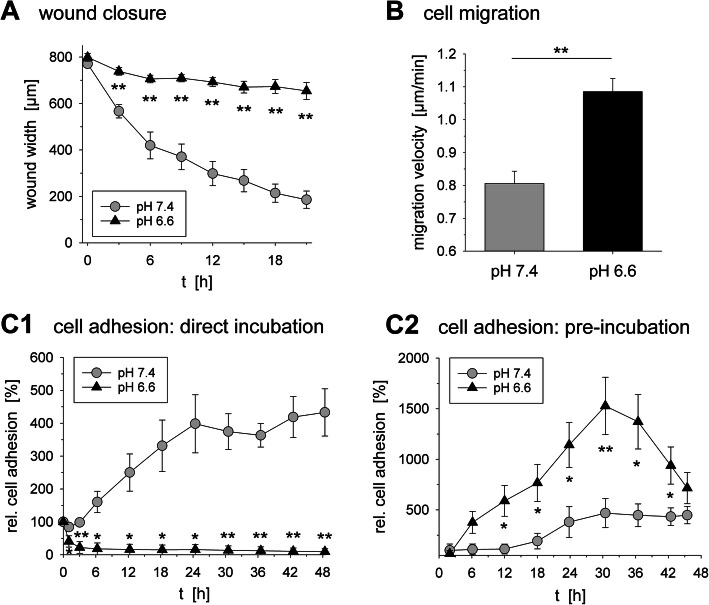
(**a**) Wound closure determined by wound width (scratch assay; *n* = 15–16) and (**b**) migration velocity (time lapse microscopy after 24 h incubation; *n* = 46) of AT1 cells kept at pH 7.4 or. (**c**) Relative adhesion of AT1 cells up to 48 h. Cells were analyzed either during continuous direct incubation at pH 6.6 for 48 h (**C1** direct incubation; *n* = 3–4) or after 24 h pre-incubation at pH 6.6 after which the medium was changed to pH 7.4 for the following 48 h (**C2** pre-incubation; *n* = 6). (*) p < 0.05, (**) p < 0.01 vs. pH 7.4

The migratory speed of AT1 cells was 40% higher at pH 6.6 compared to control conditions (Fig. 5b, Suppl. Fig. S5B). In a scratch-assay with AT1 cells the wound width at pH 7.4 was 50% after 6 h (Fig. 5a, Suppl. Fig. S5A) whereas at pH 6.6 the wound was closed by only 12%. However, even the scratch assay was performed in serum-free medium, the results may also reflect the proliferative activity of the cells.

Cell adhesion of AT1 cells was measured by impedance technique. In order to For mimic the intratumoral acidic situation, adhesion was determined during incubation of cells at low pH. At pH 6.6 AT1 cell adhesion was strongly decreased whereas cells were progressively adherent at pH 7.4 (Fig. 5C1). For checking the metastatic potential of circulating tumor cells, cells were pre-incubated at low pH, transferred to control pH and the adhesion was measured. In this setting cells incubated at pH 6.6 adhered much stronger than under control conditions (Fig. 5C2).

**Fig. 6:**
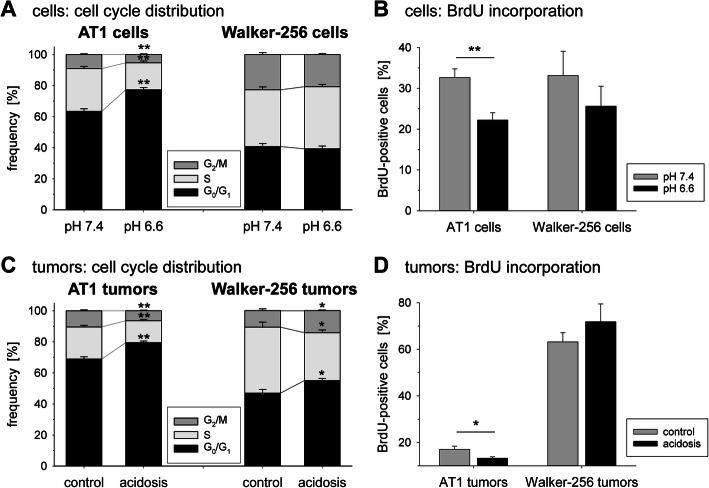
Cell cycle distribution (**a** + **c**) and fraction of actively proliferating cells (**b** + **d**) in AT1 and Walker-256 cells in vitro (**a** + **b**) and AT1 and Walker-256 tumors in vivo (**c** + **d**) after 24 h under acidotic or control conditions. n = 6–37; (*) p < 0.05, (**) p < 0.01 acidosis vs. control

Tumor cell proliferation, measured by cell cycle analyses and BrdU incorporation, showed a pH dependent cell line-specific effect of acidosis. In AT1 cells a marked G1 arrest in the cell cycle was seen at pH 6.6, whereas in Walker-256 cells the cell cycle distribution was almost independent from the extracellular pH (Fig. 6a). The number of actively DNA synthesizing cells (BrdU-positive) was significantly lower at pH 6.6 in AT1 cells and slightly reduced in Walker-256 cells (Fig. 6b). In experimental tumors in vivo an acidification of the extracellular space by forcing glycolytic metabolism led to a significant increase of cells in the G0/G1 phase and a reduced number of S phase cells in both tumor models (Fig. 6c). In AT1 tumors proliferation was lower under acidic conditions whereas cell division of Walker-256 tumors, which show a much higher baseline proliferation than AT1 tumors, was independent from an additional acidification of the tissue (Fig. 6d).

**Fig. 7:**
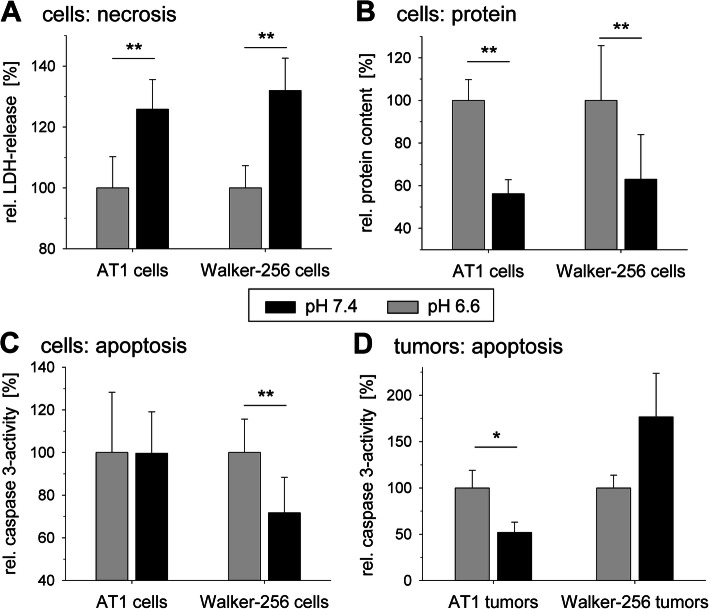
(**a**) Necrosis (relative LDH release), (**b**) cell protein and (**c**) apoptosis (relative caspase-3 activity) of AT1 and Walker-256 cells in vitro after 24 h at pH 7.4 and 6.6. (**d**) Apoptosis in AT1 and Walker-256 tumors in vivo 24 h after inducing metabolic acidosis by intensifying glycolytic metabolism. *n* = 9–15; (*) p < 0.05, (**) p < 0.01 acidosis vs. control

Furthermore, the necrotic cell death was significantly increased at pH 6.6 (Fig. 7a) leading to a reduced number of surviving cells quantified by the amount of cellular protein (Fig. 7b). Caspase-dependent apoptosis was reduced by 30% at low pH only in Walker-256 cells but not in the AT1 cell line (Fig. 7c). In solid tumors in vivo this finding was reversed showing a 50% reduction of apoptosis in AT1 tumors but not in Walker-256 tumors (Fig. 7d).

The analysis of cellular respiration showed a clear inverse relation of the baseline O_2_ consumption when lowering the extracellular pH (Suppl. Fig. S6). The O_2_ consumption rate increased by 40% when pH_e_ was reduced from 7.4 to 6.0. In normal rat kidney fibroblasts as a model of non-tumorous cells, oxygen consumption was independent from the pH (data not shown). The same pH dependency as seen for the baseline consumption (at least by trend) was found after inhibiting complex V ATP synthase (by oligomycin), after uncoupling oxygen consumption from ATP production (by FCCP) and after inhibiting complexes I and III (by rotenone + antimycin A). These results indicate that the mitochondrial O_2_ consumption as well as the non-mitochondrial O_2_ demand is pH-dependent in tumor cells.

## Discussion

In this study the impact of reduced extracellular pH in tumors on gene expression and function was analyzed. Since the aim was to find cell line-independent effects of tumor acidosis two different cell lines (AT1 prostate carcinoma and Walker-256 mammary carcinoma) were analyzed in vitro and in vivo and only consistent changes in the same direction may indicate generalized effects of an acidic tumor environment. Starting from a screening approach using NGS several genes were identified to be pH-dependently regulated in both cell lines. In order to test whether these pH-dependent genes may play a role for functional properties of tumor cells, such as proliferation, migration, cell adhesion or mitochondrial activity, a bioinformatic analysis (gene ontology overrepresentation test) was performed (Suppl. Tab. S2). It revealed that acidosis-dependent genes in AT1 or Walker-256 cells and/or tumors are associated with processes like DNA replication, DNA repair, response to cytokines, cell signaling (e.g., ERK1/2), metabolism or redox homeostasis which may play a role for the malignant potential of tumors. However, since many other aspects of the tumor cells (like differentiation, grade of malignancy etc.) may also be important for gene expression, it would also be interesting to analyze cell lines with the same genetic background but for instance different differentiation. This aspect has to be addressed in further studies.

For several genes found in the NGS analyses the pH-dependent mRNA expression was confirmed by qPCR with upregulation of Txnip, Gls2, Aox1, Tlr5, Ikbke, Per3 and downregulation of Acat2, Dnajc25, Clspn, Mmd (Fig. 1a). The upregulation of these genes was not seen during hypoxia, however, the combination of acidosis+hypoxia indicated that upregulation resulted from the reduced pH. In experimental tumors in vivo Txnip and Aox1 were consistently upregulated in both models by low tissue pH due to intensified glycolytic metabolism. However, for interpretation of the in vivo results it should kept in mind that especially in AT1 tumors the tissue already shows pronounced hypoxia even under control conditions [45] which was further enhanced by inspiratory hypoxia. Thus, changes of protein expression may not only be induced by intensified tumor acidosis but also by low pO_2_. Therefore, in hypoxic AT1 tumors it might be more suitable to compare the in vivo tumor results with the cell results obtained under combined acidosis+hypoxia (Suppl. Fig. S4). In addition, since the measurements were performed in tumor tissue lysates, the data do not only result from tumor cells but also from other cells of the tumor matrix (e.g., fibroblasts or immune cells) which also change their gene and protein expression pattern by acidosis [13]. Taking the in vitro and in vivo results together, it was shown that Txnip, Aox1, Per3 and Ypel3 were consistently upregulated in both tumor lines and Acat2, Clspn and Ercc6l were downregulated. Lamp2 has been described to be overexpressed in aggressive tumors and to be upregulated in acidosis-adapted tumor cells [34]. This protein has been proposed as a histologic marker for pathologic correlation with in vivo pH_e_ mapping [46]. However, in our study Lamp2 was found to be upregulated only in Walker-256 cells and in AT1 tumors (Figs. 1a and Fig. 3A). From these data it might be possible that acidosis-induced Lamp2 expression is cell line-specific and in solid tumors also affected by other cell types (e.g., fibroblasts). Also the duration of exposure to an acidic environment plays a role since in chronically acidosis-adapted AT1 cells an induction of Lamp2 expression was seen (Suppl. Fig. S8B).

Xu et al. [47] analyzed the expression of genes relevant for intracellular acid-base homeostasis in different tumor entities and detected numerous genes of H^+^-ATPases, MCT-, HCO_3_^−^- and NHE-transporters and some isoforms of the carbonic anhydrase to be upregulated in tumors. In our study we only found the V-ATPase gene Atp6v1c2 to be consistently upregulated upon low pH incubation. Additionally, acidosis-dependent changes of protein expression described for the AT1 cell line (e.g., NBC3, CRABP2) [48] were found in our study only in the prostate carcinoma cell line but not in the Walker-256 mammary carcinoma line suggesting a cell line-specific response.

The most prominent acidosis-dependent changes in the expression were observed for Txnip, encoding the thioredoxin interacting protein (TXNIP), which was increased in both cell lines under in vitro and in vivo conditions. This protein is part of the redox system of tumors and inhibits cytosolic thioredoxin Trx-1 [49]. Since elevated levels of Trx-1 are associated with tumor progression the increase of TXNIP protein was associated with a higher overall survival of patients with mammary carcinomas [50] suggesting that TXNIP could inhibit tumor progression [49]. An increased Txnip expression with decreasing pH_e_ has also been described by others [39, 51]. High TXNIP levels were associated with a glycolytic phenotype of tumors [51] but also hypoxia downregulated TXNIP expression [52]. Obviously, the interaction of different environmental parameters plays an important role which is also seen in the present study, when cells were exposed to simultaneous acidosis+hypoxia leading to no changes in Txnip expression (Fig. 1C).

The situation becomes even more complex because acidosis induces the expression of genes which reduce the oxidative stress in tumors. GLS2, which is an important enzyme in conversion of glutamine to glutamate, and thereby a regulator of glutathione (GSH) synthesis [38], is upregulated in tumor cells at low pH (Figs. 1 and 2). Therefore, acidosis can either reduce the anti-oxidative capacity (e.g., via Txnip expression) or increase it (e.g., via Gls2 expression). In the present study in both cell lines low pH_e_ led to an increased necrotic cell death (Fig. 7a, b) which may support the hypothesis of a reduced anti-oxidative capacity. One mechanism by which acidosis may induce the Gls2 expression is an upregulation of p53 [38]. Even though an increase of p53 at the mRNA level was not seen in our study, p53 might be involved on the function level. Since p53 is able to induce a G1 cell cycle arrest [53] the observed changes of the cell cycle distribution in both tumor lines (Fig. 6) could reflect a p53-dependent mechanism.

In an additional experiment the impact of long-term acidosis (5 weeks) was compared to acute changes after 24 h at low pH was analyzed. The comparison of acute and chronic changes revealed that several genes were regulated in the same direction, for instance Per3. Per3 expression was doubled not only after 24 h in both cell lines (Fig. 1a) but also after 5 weeks (Suppl. Fig. S8B). Therefore, Per3 seems to be an uniformly regulated gene during acute and chronic acidosis. Another gene, which has been described to be upregulated by long-term acidosis, is the carbonic anhydrase IX (Ca9) [54]. This was also seen in the present data, however in a time-dependent degree. During acute acidosis Ca9 expression was increased by a factor of 2.6 ± 0.5 whereas after chronic adaptation the expression was higher by a factor of 4.2 ± 0.1. Finally, some genes were differentially regulated by acute or chronic acidosis. For instance, Lamp2 was not regulated after 24 h in AT1 cells (Fig. 1a) but was significantly upregulated in the same cell line after 5 weeks (Suppl. Fig. S8B) which is in accordance with results by others [34]. Since perfusion, and by this the oxygen supply of the tumor tissue, can vary in a time scale of minutes to hours [55], the impact of both short- and long-term pH changes on gene expression and function should be taken into account.

The results of the present study also clearly indicate that low extracellular pH strongly affects migration and cell adhesion of tumor cells (Fig. 5). These findings may also be related to the observed changes in gene expression. An increased expression of Per3 [40] or Ikbke [41] is associated with increased migratory potential. An elevated Txnip level has been shown to reduce the adhesion of tumor cells [43]. However, on the other hand elevated Txnip levels has been shown to inhibit migration [42]. The overall effect of extracellular acidosis on migration is difficult to predict. The results of the adhesion measurements in vitro (Fig. 5c) are in good accordance with previous in vivo experiments using the same AT1 tumor model, in which pre-conditioned tumor cells led to a higher number of lung metastases when injected i.v [7].

Measuring the cellular oxygen consumption clearly showed that in the AT1 tumor cells mitochondrial respiration increased with decreasing pH (Suppl. Fig. S6). Even under control condition AT1 cells show an almost maximal O_2_ consumption rate which could not be raised by collapsing the H^+^-gradient (incubation with FCCP). The baseline and the maximum O_2_ consumption increased almost linearly with decreasing pH. ATP-linked respiration (oligomycin incubation) and non-mitochondrial O_2_ consumption increased only slightly at low pH. Such a pH-dependent increase of oxidative phosphorylation has also been described by others [56, 57]. However, some authors described a decrease of O_2_ consumption at pH 6.3–6.5 [58, 59]. These contradictory results may be attributed to tumor cell-specific differences in the metabolic pathways (e.g., glycolysis, glutaminolysis, fatty acid metabolism) fueling the TCA [56]. The changes seen in the present study could be, at least partially, the results of the changed protein expression by acidosis. For cardiomyocytes it has been shown that a reduced TXNIP expression reduces the oxygen consumption rate [44] which would be in accordance to the present study.

## Conclusions

In conclusion, our study demonstrates that the acidic extracellular pH in tumors plays an important role for gene expression independently from tissue hypoxia. In parallel, this study shows that acidosis affects several functional properties of tumors such as proliferation, cell cycle distribution, migration, or cell death and therefore for the malignant potential of tumors. Since several of the genes found to be pH-dependent directly or indirectly interfere with the functional parameters studied, it can be hypothesized that acidosis modulates tumor cell function via pH-dependent genes. However, this hypothesis of an causative link has to be addressed in further studies. The study also demonstrates that, with respect to gene expression, the results of cell culture experiments cannot be directly transferred to the in vivo situation were other cell types interfere with the tumor cells and form a micromilieu in which different metabolic parameters, cell-cell- or cell-matrix-contacts, cytokines or other paracrine factors play a relevant role.

## Supplementary Information

**Additional file 1. **Additional material (tables and figures) is provided in the “Additional file 1“. **Tab. S1.** Primers used for qPCR. **Tab. S2.** Gene ontology analysis for acidosis-regulated genes in cells and tumors. **Fig. S1.** Venn diagrams of genes regulated by acidosis. **Fig. S2.** Comparison of NGS results in vitro and in vivo. **Fig. S3.** mRNA expression after 48 h acidosis. **Fig. S4.** Comparison of the impact of acidosis in vitro and in vivo. **Fig. S5.** Wound closure and migration distance during acidosis. **Fig. S6.** Cellular oxygen consumption during acidosis. **Fig. S7.** Tumor cell proliferation during long-term acidosis. **Fig. S8.** Gene expression of tumor cells chronically adapted to acidosis. The raw data of the NGS analyses are available via: https://www.ncbi.nlm.nih.gov/geo/query/acc.cgi?acc=GSE162705

## Data Availability

All data generated or analyzed during this study are included in this published article and it supplementary material file. The datasets used and/or analysed during the current study are available from the corresponding author on reasonable request.

## Abbreviations

AA: Antimycin A
Acat2: Acetyl-CoA acetyltransferase 2
Aox1: Aldehyde oxidase 1
Brip1: BRCA1 interacting protein C-terminal helicase 1
Clspn: Claspin
Crem: cAMP responsive element modulator
Dnajc25: DnaJ heat shock protein family (Hsp40) member C25
Ercc6l: ERCC excision repair 6 like, spindle assembly checkpoint helicase
Gls2: Glutaminase 2
Gstp1: Glutathione S-transferase pi 1
Ikbke: Inhibitor of kappa light polypeptide gene enhancer in B-cells, kinase epsilon
Lamp2: Lysosomal associated membrane protein 2
MIBG: Meta-iodobenzylguanidine
Mmd: Monocyte to macrophage differentiation associated
Per3: Period circadian clock 3
Pink1: PTEN induced kinase 1
Rif1: Replication timing regulatory factor 1
Tlr5: Toll-like receptor 5
Txnip: Thioredoxin interacting protein
Ypel3: Yippee like 3
Zmpste24: Zinc metallopeptidase STE24
