# Supplementary material for: Impact of the acidic environment on gene expression and functional parameters of tumors in vitro and in vivo
Source: J Exp Clin Cancer Res. 2021 Jan 6;40:10. doi: 10.1186/s13046-020-01815-4 (PMC7786478; doi:10.1186/s13046-020-01815-4)
Supplement: Supplementary file 1 — Additional file 1. Additional material (tables and figures) is provided in the “Additional file 1“. Tab. S1. Primers used for qPCR. Tab. S2. Gene ontology analysis for acidosis-regulated genes in cells and tumors. Fig. S1. Venn diagrams of genes regulated by acidosis. Fig. S2. Comparison of NGS results in vitro and in vivo. Fig. S3. mRNA expression after 48 h acidosis. Fig. S4. Comparison of the impact of acidosis in vitro and in vivo. Fig. S5. Wound closure and migration distance during acidosis. Fig. S6. Cellular oxygen consumption during acidosis. Fig. S7. Tumor cell proliferation during long-term acidosis. Fig. S8. Gene expression of tumor cells chronically adapted to acidosis. The raw data of the NGS analyses are available via: https://www.ncbi.nlm.nih.gov/geo/query/acc.cgi?acc=GSE162705 [file 13046_2020_1815_MOESM1_ESM.pdf]

## Impact of the acidic environment on gene expression and functional parameters of tumors *in vitro* and *in vivo*

Mandy Rauschner, Luisa Lange, Thea Hüsing, Sarah Reime, Alexander Nolze, Marcel Maschek, Oliver Thews, Anne Riemann

Institute of Physiology, University Halle, Magdeburger Str. 6, 06112 Halle (Saale), Germany

## Supplementary Material

**Table S1:** Primers used for quantitative PCR.

| Target | forward primer | reverse primer |
|---|---|---|
| *Acat2* | GACTGGAACGGTCACCCCAG | TCGGTCCTACTCCCATGACA |
| *Aox1* | GAGCAGACATCTGGACTCGG | GTCAGGACACTTGCGGAGAA |
| *App* | GAGGACTGACCACTCGACCA | GAGGACTGACCACTCGACCA |
| *Brip1* | CTCAGGGGTCCCGATGACTA | CTCCCGAGGCTGACAAGTTC |
| *Calcoco1* | CAAACCCAGCAGAAAGTGGC | TTCGGTCTCTAGCTCCTGCT |
| *Clspn* | AGCCAAGTGGAGAAAGGAGC | GGAAGAAGAGTTACCTCCCGC |
| *Crem* | TCCGAGCTCCTACTACTGCT | TTTCATCAGCCTCAGCTCCC |
| *Dnajc25* | GTCAGCGTGTGTGCCATTTC | TAGACCCACCGGCAATACCA |
| *Ercc6l* | GTTTAGCGGAAGTGGGGACT | CCAACTGCTCCAAAGCTTCC |
| *Fstl1* | TGCCCTCATTGAACTGTCCG | ACAGGAACAGACACAGCGAT |
| *Fundc1* | GGCTGGTGTGCAGGATTTTT | GTGCTGCCTTATTTGCTCGC |
| *Gls2* | CCTTTTCCTTTAGATGTGGGGC | AAGCAGGTCACCAAGTCGAG |
| *Gstp1* | TGTACTTCCCAGTTCGAGGG | CGAGCCTTGAAGCCAGACAT |
| *Ikbke* | ACCTGTAACTCAGAAGCCCG | TCCTGCATGTGGAAGACCAG |
| *Il6r* | CCTATACCCCTGCCCACATTC | TCAGCGGTCCCAAGGGATAC |
| *Lamp2* | GGTGGTTTCCGTGTCTCGAA | TCCAGTATGATGGCGCTTGA |
| *Ltbp2* | GATATGCTGTTGCAGCCGTG | CACCTTTGTCAGGGAGGGTC |
| *Mmd* | TGCTACACACACGCATTCCT | CCCATCCCGTAGATCCATGC |
| *Per3* | GAACAGGAAACAACCGCACC | CCACGGGCTTGAATCCTTCT |
| *Pink1* | TGTATGAAGCCACCATGCCC | GCTTAAGATGGCTTCGCTGG |
| *Rif1* | CACTGCGCTAGAAATGGGGA | TGTCCCACTACGGAAGCCTA |
| *Tlr5* | CTGTCTGACCTCAAGCGTGT | GGGCCACCTCAAATACTGCT |
| *Txnip* | CCAGCCTACAGGTGAGAACG | GGCTGGGACGATCGAGAAAA |
| *Ypel3* | GCGTGCCTACCTCTTCAACT | TTGCAGTTCTCGCAGTGGAT |
| *Zmpste24* | ACAACGACTCATGTACCACCA | CCGAAGCCAGCAGAACTACA |

| **Housekeeper** | | |
|---|---|---|
| *Hprt1* | ACCAGTCAACGGGGGACATA | TTGGGGCTGTACTGCTTGAC |
| *18S* | CTGAGAAACGGCTACCACATC | CCCAAGATCCAACTACGAGC |

**Table S2A:** PANTHER Gene Ontology Overrepresentation Test for biological processes and molecular functions in acidosis-regulated genes in AT1- and Walker-256 cells *in vitro*.

| Gene function of acidosis regulated genes in cells (*in vitro*) | |
|---|---|
| Biological Process | Molecular Function |
| • cellular iron ion homeostasis (GO:0006879)<br>• cellular transition metal ion homeostasis (GO:0046916)<br>• transition metal ion homeostasis (GO:0055076)<br>• metal ion homeostasis (GO:0055065)<br>• cation homeostasis (GO:0055080)<br>• ion homeostasis (GO:0050801)<br>• homeostatic process (GO:0042592)<br>• cellular metal ion homeostasis (GO:0006875)<br>• cellular cation homeostasis (GO:0030003)<br>• cellular ion homeostasis (GO:0006873)<br>• cellular chemical homeostasis (GO:0055082)<br>• cellular homeostasis (GO:0019725)<br>• iron ion homeostasis (GO:0055072)<br><br>• nucleosome assembly (GO:0006334)<br><br>• glutathione metabolic process (GO:0006749)<br>• cellular modified amino acid metabolic process (GO:0006575)<br>• sulfur compound metabolic process (GO:0006790)<br>• cofactor metabolic process (GO:0051186)<br><br>• autophagy of mitochondrion (GO:0000422)<br>• mitochondrion organization (GO:0007005)<br><br>• transposition (GO:0032196)<br><br>• maintenance of location (GO:0051235) | • ferrous iron binding (GO:0008198)<br>• iron ion binding (GO:0005506)<br>• transition metal ion binding (GO:0046914)<br>• ion binding (GO:0043167)<br><br>• single-stranded RNA binding (GO:0003727)<br><br>• oxidoreductase activity (GO:0016491)<br>• catalytic activity (GO:0003824)<br><br>• transmembrane signaling receptor activity (GO:0004888)<br>• signaling receptor activity (GO:0038023) |

**Table S2B:** PANTHER Gene Ontology Overrepresentation Test for biological processes and molecular functions in acidosis-regulated genes in AT1- and Walker-256 tumors *in vivo*.

| Gene function of acidosis regulated genes in tumors (*in vivo*) | |
|---|---|
| Biological Process | Molecular Function |
| • pre-replicative complex assembly involved in nuclear cell cycle DNA replication (GO:0006267)<br>• protein-DNA complex assembly (GO:0065004)<br>• protein-DNA complex subunit organization (GO:0071824)<br>• nuclear DNA replication (GO:0033260)<br>• cell cycle DNA replication (GO:0044786)<br>• DNA-dependent DNA replication (GO:0006261)<br>• DNA replication (GO:0006260)<br><br>• mitotic DNA replication (GO:1902969)<br><br>• double-strand break repair via break-induced replication (GO:0000727)<br>• double-strand break repair via homologous recombination (GO:0000724)<br>• double-strand break repair (GO:0006302)<br>• DNA repair (GO:0006281)<br>• response to stress (GO:0006950)<br>• DNA metabolic process (GO:0006259)<br>• recombinational repair (GO:0000725)<br>• DNA recombination (GO:0006310)<br><br>• chemokine-mediated signaling pathway (GO:0070098)<br>• cellular response to chemokine (GO:1990869)<br>• response to chemokine (GO:1990868)<br>• response to cytokine (GO:0034097)<br>• response to organic substance (GO:0010033)<br>• cellular response to cytokine stimulus (GO:0071345)<br>• cytokine-mediated signaling pathway (GO:0019221)<br><br>• granulocyte chemotaxis (GO:0071621)<br>• leukocyte chemotaxis (GO:0030595)<br>• cell chemotaxis (GO:0060326)<br>• leukocyte migration (GO:0050900)<br>• myeloid leukocyte migration (GO:0097529)<br><br>• DNA replication initiation (GO:0006270)<br><br>• lymphocyte migration (GO:0072676)<br><br>• response to interleukin-1 (GO:0070555)<br><br>• cellular response to tumor necrosis factor (GO:0071356) | • DNA replication origin binding (GO:0003688)<br><br>• single-stranded DNA binding (GO:0003697)<br><br>• catalytic activity, acting on DNA (GO:0140097)<br>• catalytic activity (GO:0003824)<br><br>• cytokine activity (GO:0005125)<br><br>• cytokine receptor binding (GO:0005126)<br><br>• ATPase activity, coupled (GO:0042623)<br><br>• G protein-coupled receptor binding (GO:0001664) |

| | |
|---|---|
| • response to tumor necrosis factor (GO:0034612)<br><br>• positive regulation of ERK1 and ERK2 cascade (GO:0070374)<br>• regulation of ERK1 and ERK2 cascade (GO:0070372)<br><br>• ERK1 and ERK2 cascade (GO:0070371)<br><br>• inflammatory response (GO:0006954)<br>• defense response (GO:0006952)<br><br>• DNA biosynthetic process (GO:0071897)<br><br>• response to lipopolysaccharide (GO:0032496)<br>• response to molecule of bacterial origin (GO:0002237)<br>• response to other organism (GO:0051707)<br>• response to external biotic stimulus (GO:0043207)<br>• response to biotic stimulus (GO:0009607)<br><br>• carboxylic acid biosynthetic process (GO:0046394)<br>• carboxylic acid metabolic process (GO:0019752)<br>• oxoacid metabolic process (GO:0043436)<br>• organic acid metabolic process (GO:0006082)<br>• organic acid biosynthetic process (GO:0016053)<br>• small molecule biosynthetic process (GO:0044283)<br>• cellular amino acid metabolic process (GO:0006520) | |

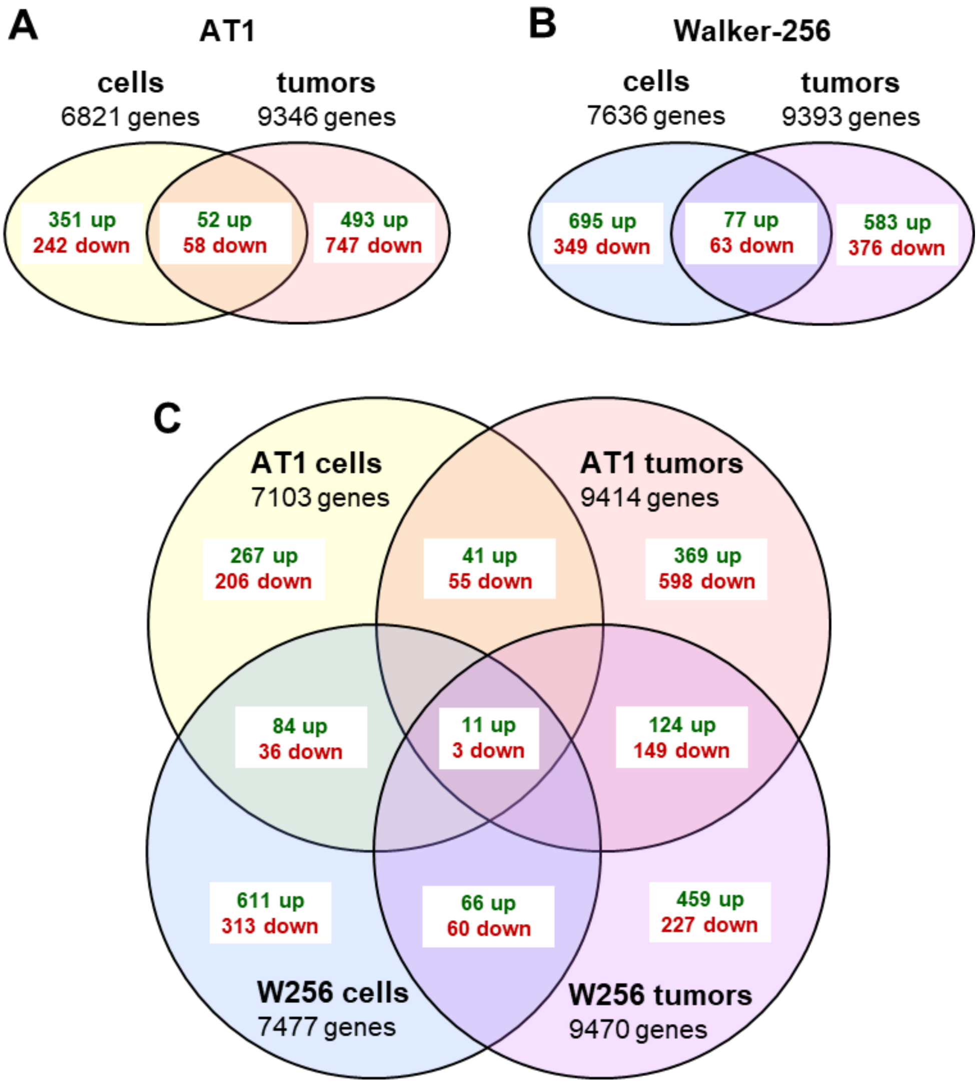


**Fig. S1:** Venn diagrams of the number of genes regulated by acidosis in (**A**) AT1 cells and tumors, (**B**) Walker-256 cells and tumors and (**C**) combined both cell lines *in vitro* (cells) and *in vivo* (tumors) measured by NGS. The total number of genes detectable was defined by an abundance >10 FPM. Up- or down-regulation of genes was defined in cells by an expression change of +/- 1.5 and in tumors by +/- 1.75. cells: n=3; tumors n=4.

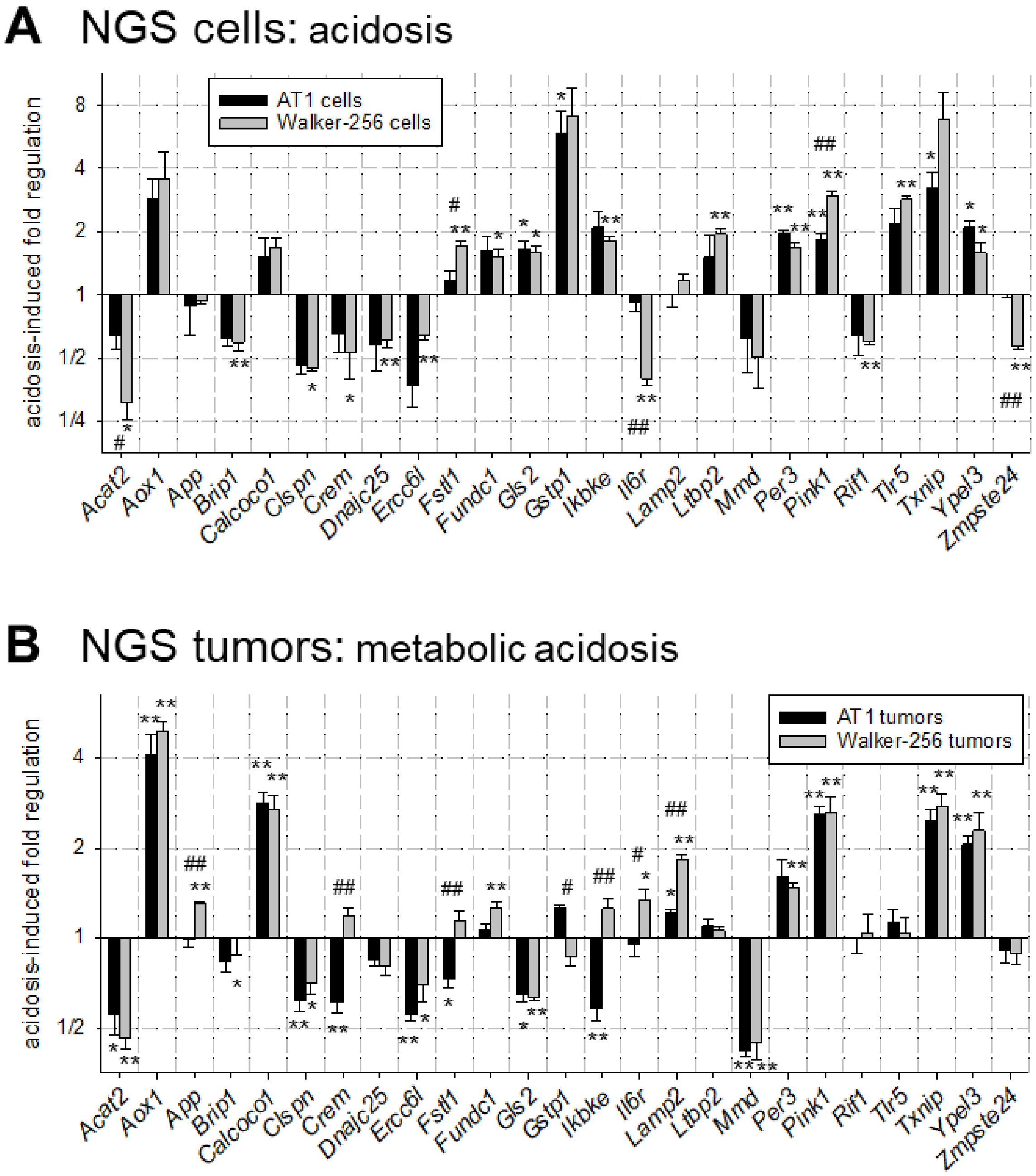


**Fig. S2:** Change of RNA expression of target genes measured by NGS induced by acidosis (corresponding to the qPCR analyses shown in Figs. 1 and 2). AT1 and Walker-256 cells were incubated 24 h at pH 6.6 (room air), whereas in AT1 and Walker-256 tumors *in vivo* metabolic acidosis was induced for 24 h by forcing glycolytic metabolism. n=3-4; (*) $p<0.05$, (**) $p<0.01$ vs. pH 7.4/control; (#) $p<0.05$, (##) $p<0.01$ AT1 vs. Walker-256 cells.

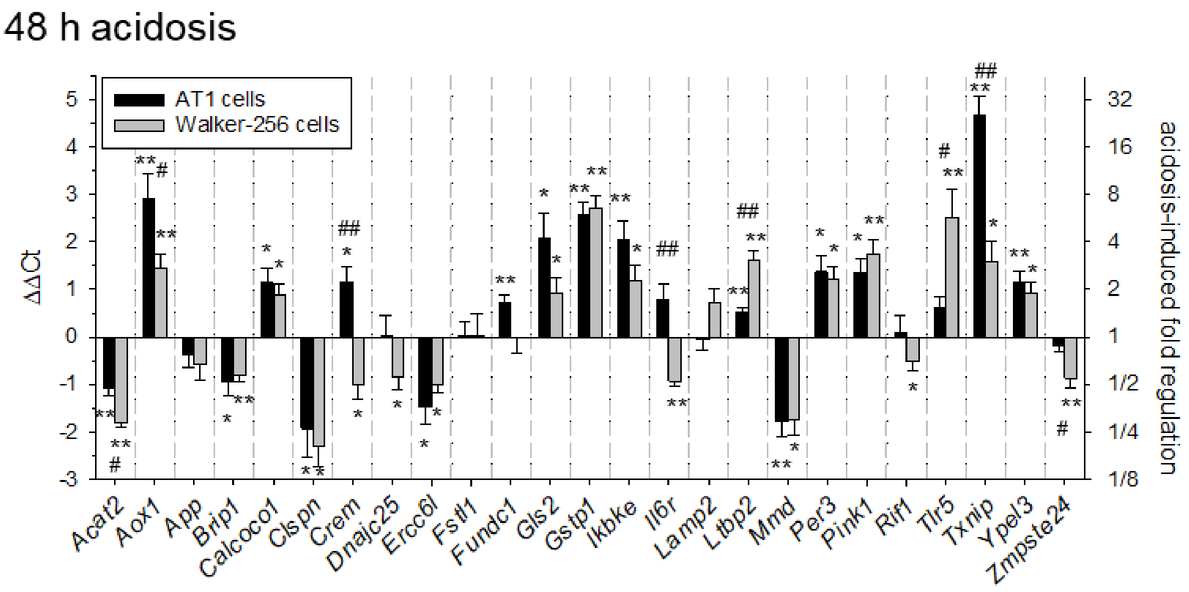


**Fig. S3:** mRNA expression of target genes (measured by qPCR) in AT1 prostate and Walker-256 mammary carcinoma cells after 48 h under acidic (pH 6.6, room air) conditions. n=4-10; (*) $p<0.05$, (**) $p<0.01$ vs. control; (#) $p<0.05$, (##) $p<0.01$ AT1 vs. Walker-256 cells.

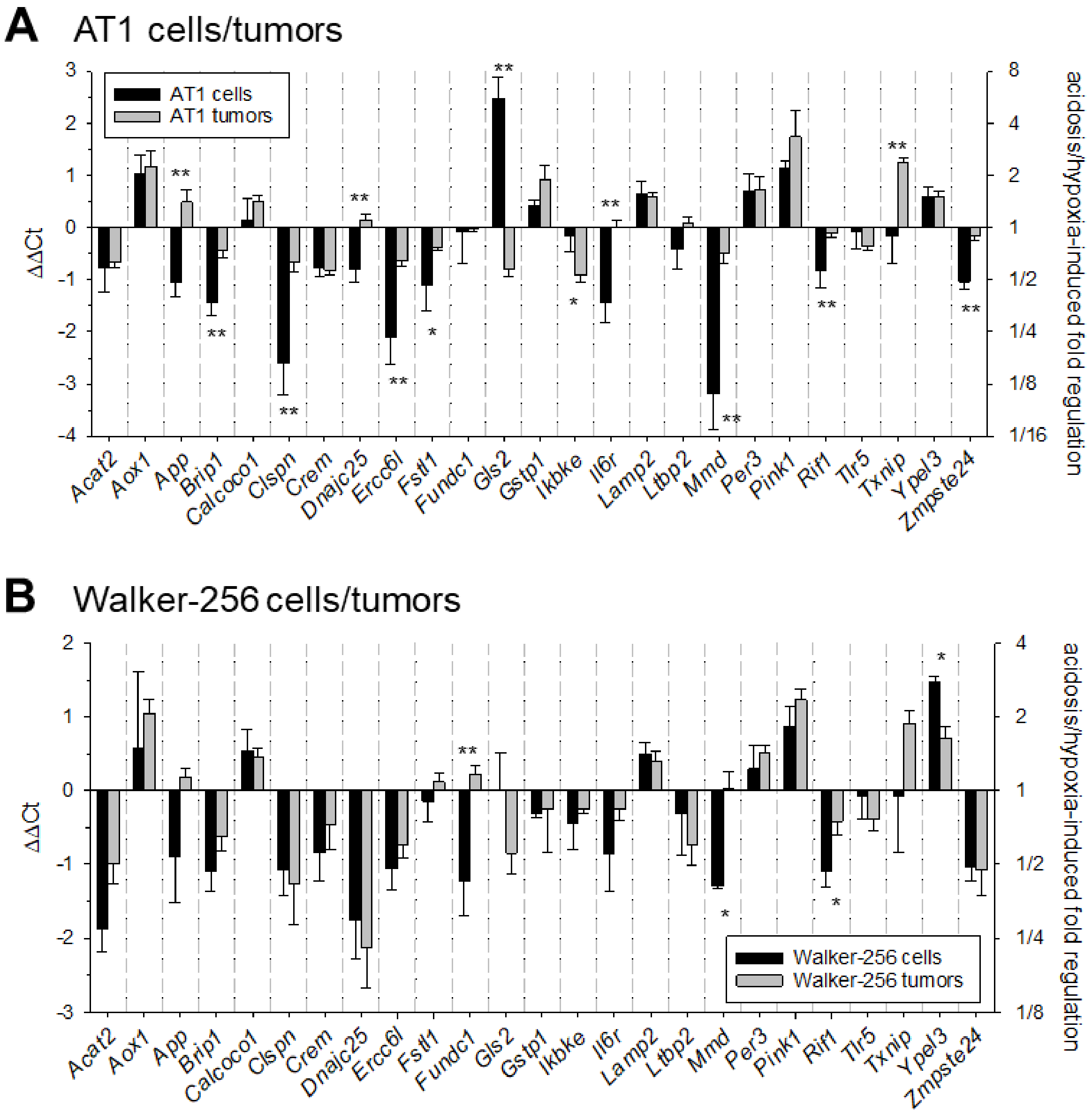


**Fig. S4:** Comparison of the impact of combined hypoxia+acidosis on mRNA expression (measured by qPCR) *in vitro* (isolated cells) and *in vivo* (experimental tumors) of the (**A**) AT1 and (**B**) Walker-256 cell line. n = 4–10 (cells), n = 4–22 (tumors); (*) $p < 0.05$, (**) $p < 0.01$ cells *vs.* tumors.

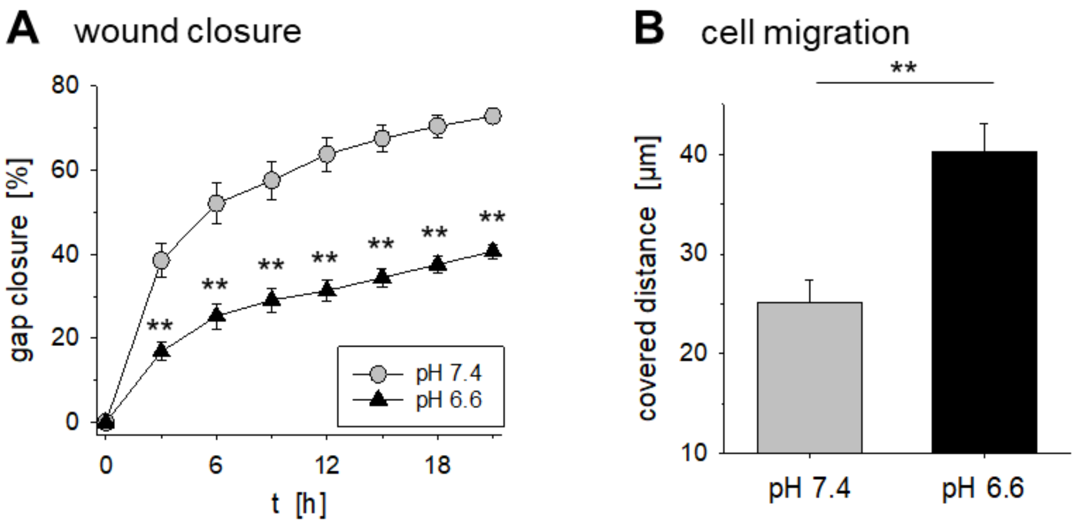


**Fig. S5:** (**A**) Wound closure determined by percent gap closure (scratch assay; n=15-16) and (**B**) covered migration distance (time-lapse microscopy after 24 h incubation; n=46) of AT1 cells kept at pH 7.4 or 6.6. (**) $p<0.01$ *vs.* pH 7.4.

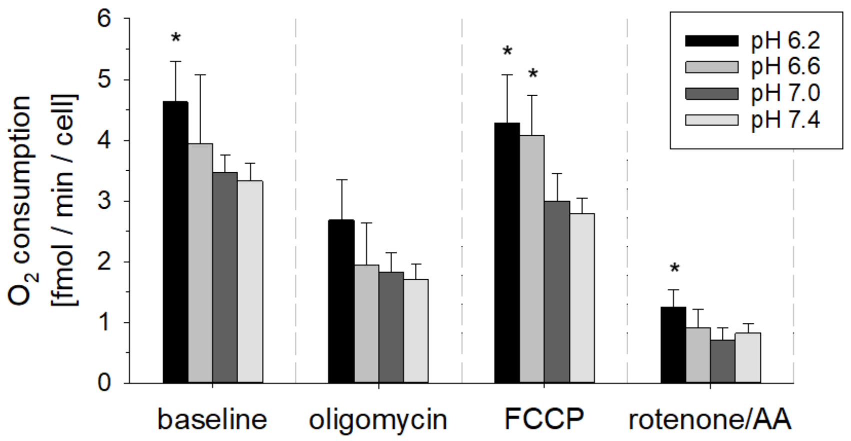


**Fig. S6:** Cellular oxygen consumption of AT1 cells after incubation at different pH for 3 h. Besides baseline conditions, $O_2$ consumption was measured after adding oligomycin (inhibiting complex V ATP synthase), carbonyl cyanide-4-(trifluoromethoxy) phenylhydrazone (FCCP; uncoupling oxygen consumption from ATP production) and rotenone + antimycin A (inhibiting complexes I and III). n = 6–8; (*) $p < 0.05$ *vs.* pH 7.4.

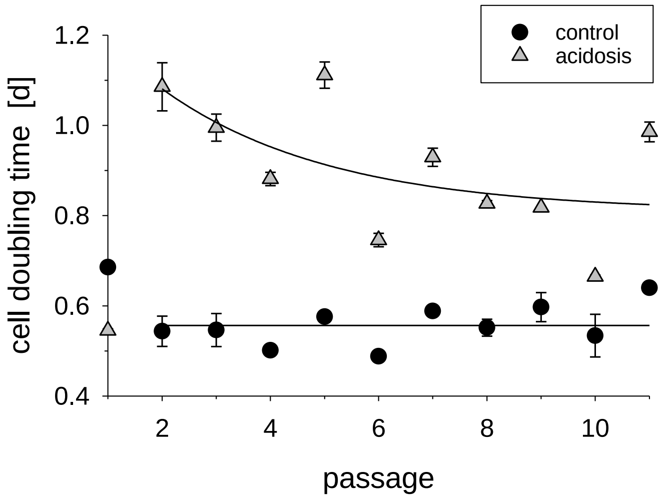


**Fig. S7:** AT1 tumor cell proliferation (expressed by the cell doubling time) in control cells (pH 7.4) and during long-term adaptation to extracellular acidosis (pH 6.6) for up to 11 passages (5 weeks). Cells were passaged every 3 or 4 days. n = 2–4.

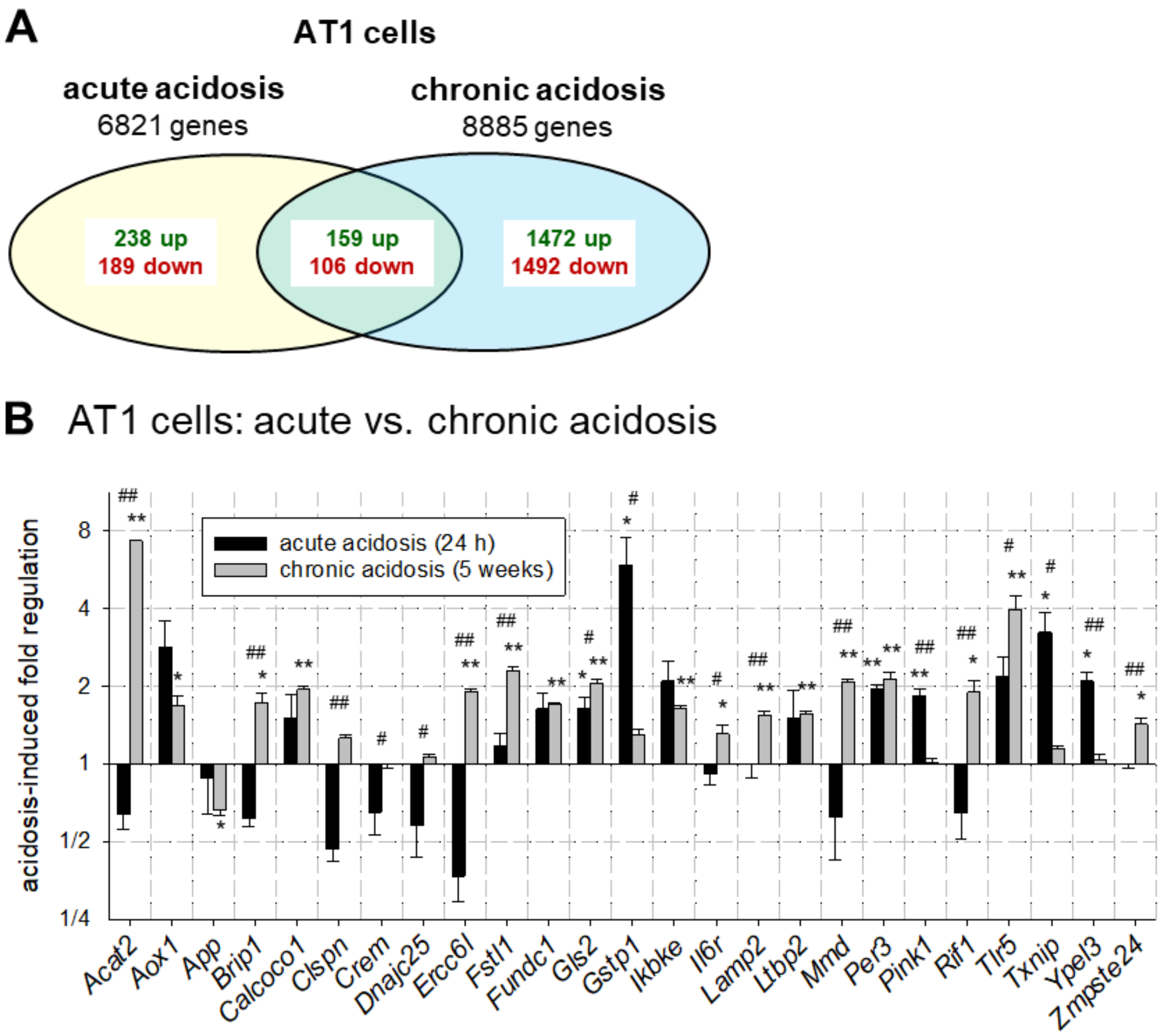


**Fig. S8:** (**A**) Venn diagram of number of genes regulated in AT1 cells by acute (24 hours) or chronic (5 weeks) acidosis measured by NGS. The total number of genes detectable was defined by an abundance >10 FPM. Up- or down-regulation of genes was defined by an expression change of +/- 1.5. n=3-4. (**B**) Change of RNA expression of target genes measured by NGS in AT1 cells induced by acute (24 hours) or chronic (5 weeks) acidosis. n=3-4; (*) p<0.05, (**) p<0.01 *vs.* pH 7.4; (#) p<0.05, (##) p<0.01 acute *vs.* chronic acidosis.
